# Carer distress among community living older adults with complex needs in the pre- and post-COVID-19 era: a national population study

**DOI:** 10.1038/s41598-022-24073-0

**Published:** 2022-11-16

**Authors:** Philip J. Schluter, Rebecca Abey-Nesbit, Annabel Ahuriri-Driscoll, Hans Ulrich Bergler, Jacqueline C. Broadbent, Michaela Glanville, Sally Keeling, Hamish A. Jamieson

**Affiliations:** 1 Te Kaupeka Oranga| Faculty of Health, Te Whare Wānanga o Waitaha| University of Canterbury, Private Bag 4800, Christchurch, 8140 New Zealand; 2 School of Clinical Medicine, Primary Care Clinical Unit, The University of Queensland, Brisbane, Australia; 3 Department of Medicine, University of Otago, Christchurch, New Zealand; 4 Older Person’s Health, Canterbury District Health Board, Christchurch, New Zealand

**Keywords:** Health care, Risk factors

## Abstract

Carer distress is one important negative impact of caregiving and likely exacerbated by the novel coronavirus disease 2019 (COVID-19) pandemic, yet little population-based epidemiological information exists. Using national data from repeated standardized comprehensive geriatric needs assessments, this study aims to: describe the pattern of caregiver distress among those providing informal care to community-living adults aged ≥ 65 years with complex needs in New Zealand over time; estimate the COVID-19 effect on this temporal pattern; and, investigate relationships between participants’ sociodemographic and selected health measures on caregiver distress. Fractional polynomial regression and multivariable multilevel mixed-effects models were employed. Overall, 231,277 assessments from 144,358 participants were analysed. At first assessment, average age was 82.0 years (range 65–107 years), and 85,676 (59.4%) were female. Carer distress prevalence increased from 35.1% on 5 July 2012 to a peak of 48.5% on 21 March 2020, when the New Zealand Government announced a national lock-down. However, the population attributional fraction associated with the COVID-19 period was small, estimated at 0.56% (95% CI 0.35%, 0.77%). Carer distress is common and has rapidly increased in recent years. While significant, the COVID-19 impact has been relatively small. Policies and services providing efficacious on-going strategies to support caregivers deserves specific attention.

## Introduction

It has been extensively documented that the world is experiencing an unprecedented accelerated ageing population, driven by increasing levels of life expectancy and decreasing levels of fertility^1^. Globally, there are now more people than ever before, and we are living longer. Many countries’ health care delivery systems have become unsustainable, needing reconceptualization and transformation^2^. An increased emphasis on disease prevention and enabling people to stay in their own homes longer, with an interconnected structure supporting them, are recurrent themes for improving sustainability^3^. Implicit in this is an increased reliance on unpaid informal carers, primarily family members including spouses, and friends^4^. However, this reliance can come at a personal cost, particularly to those caring for older adults who are experiencing increased frailty or complex needs. Currently approximately 10% of New Zealand adults are carers, many to older adults; and this percentage is likely to increase as our population ages^5^.

Informal caregiving can be a rewarding yet demanding role^6^, with both positive and negative impacts^7,8^. Documented negative caregiving sequelae are both mental and physical^8^, and include interrupted employment^9^, reduced quality of life^4,10^, poorer self-rated health^4^, increased stress^11–15^, and arguably increased mortality^16,17^. These effects can ripple further, impacting on the carer’s family, their wellbeing, and beyond^5^. The caring role is disproportionately shouldered by women^5,14^. Negative health effects from caregiving are more likely to be incurred by caregivers who are female, spousal, or who are providing intensive care^8^. Duration of caring and the dependence-level of those being cared for are important predictors of caregiver burden^10,14^.

While much is known about the effects of informal caregiving^8^, little epidemiological information exists investigating important known caregiver attributes over time. For instance, we could find no study which epidemiologically tracked population levels of caregiver distress. This is despite increased stress being widely recognised as a negative impact from caregiving^11–15^, many health care delivery providers are increasingly relying upon this unpaid workforce^4^, and mental illness becoming increasingly important and prevalent in contemporary societies^18^. Unless this crucial yet largely invisible sector group is monitored, we may miss key indicators that support their wellbeing^5^, missing opportunities for early intervention and potentially splintering their relationship with healthcare providers, creating a new unintended public health crisis, increasing societal and economic burden, and ultimately losing caregiver and community goodwill. From this perspective, caregiver distress stands out as being particularly worthy of epidemiological investigation.

Any contemporary population-based caregiver investigation measuring distress over time is likely to be disrupted by the global novel coronavirus disease 2019 (COVID-19) pandemic, declared by the World Health Organization on 11th March 2020^19^. As of 6 October 2022, 617 million confirmed cases and 6.53 million deaths globally have been reported^20^; although the true numbers are likely to be much higher, and the long term impacts on life expectancy and age-related health conditions are largely unknown. The COVID-19 pandemic has had profound and enduring global effects, and continues to dominate many governmental health, political, economic, and social agendas^21^. Prior to the pandemic, mental health disorders were already among the leading causes of global disability-adjusted life-years^18^. Prevalence of these disorders has continued to increase since this pandemic declaration^22^, and continue to increase^23^. Moreover, there is emerging evidence that the COVID-19 pandemic has worsened pre-existing psychiatric disorders, such as Obsessive–Compulsive Disorder^24,25^. Social distancing and isolation resulting in loneliness have also been shown to worsen the symptoms of anxiety and depression^26,27^. Caregivers have not been immune, experiencing increases in burden and impact since the start of the COVID-19 pandemic^28,29^. Many caregivers have also reported a reduction in behavioural changes during the pandemic associated with preventing or mitigating mental health illness^30^. In order to investigate carer distress epidemiology and the impact of the COVID-19 pandemic comprehensive data about informal care settings are required.

Since 2012, all community care recipients aged over 65 years in New Zealand have undergone a standardized comprehensive geriatric needs assessment using the Home Care International Residential Assessment Instrument (interRAI-HC)^31^. It collects information over multiple health and social domains, including informal helper status and their distress. The instrument is used with the frail elderly or persons with complex needs in home and community-based settings who are seeking or receiving formal public health care and supportive services. The instrument is principally designed to obtain person‐level information to support care plan development, including assessment questions related to receiving informal care and the perceived wellbeing or distress of those caring. InterRAI-HC information is electronically stored, held by New Zealand’s Technical Advisory Services. With approval from the Ministry of Health, consented anonymised data are released for research purposes.

Utilising this national electronic repository, this study aims to: describe the pattern of caregiver distress among those providing informal care to community-living older adults receiving a home-based interRAI-HC assessment in New Zealand over time; estimate the COVID-19 effect on this temporal pattern; and, investigate the relationship between participants’ sociodemographic and selected health measures on caregiver distress.

## Methods

### Study design

This study is a secondary analysis of routinely collected data from a continuously recruited national cohort of community living older adults with complex needs in New Zealand.

### Participants

Participants included adults aged ≥ 65 years with one or more interRAI-HC assessments undertaken between 5 July 2012 and 31 December 2020, inclusive, who consented to their data being used for planning and research purposes. Only those residing at their private home, apartment or in a rented room at assessment, and having at least one informal carer were included.

### Instrument and primary measures

The interRAI-HC 9.1 instrument (© interRAI Corporation, Washington, D.C., 1994–2009), modified with permission for New Zealand, is used under license to the Ministry of Health (www.interrai.co.nz). It is composed of 236 questions across 20 domains, and yields internationally valid and reliable scales^31^. Informal carers were defined as including family members, friends, and neighbours who provide unpaid care to the older adult. Caregiver distress was defined as recording ‘yes’ to one or more of three items within the interRAI-HC assessment: ‘informal helper(s) is unable to continue caring activities’; ‘primary informal helper expresses feelings of distress, anger, or depression’; and, ‘family or close friends report feeling overwhelmed by person’s illness’. This measure of caregiver distress has been previously employed^32–34^, and provides a broad indication of distress in the informal caregiving support system that captures both the feeling of distress and the caregiving situation in distress. It also captures the reserves of the informal caregiver support system^32^. The assessment process draws on multiple sources recorded by the assessor, and can include background information from a referrer, direct questioning, and clinical observations and judgement particularly when the primary caregiver is present during assessment.

**Figure 1:**
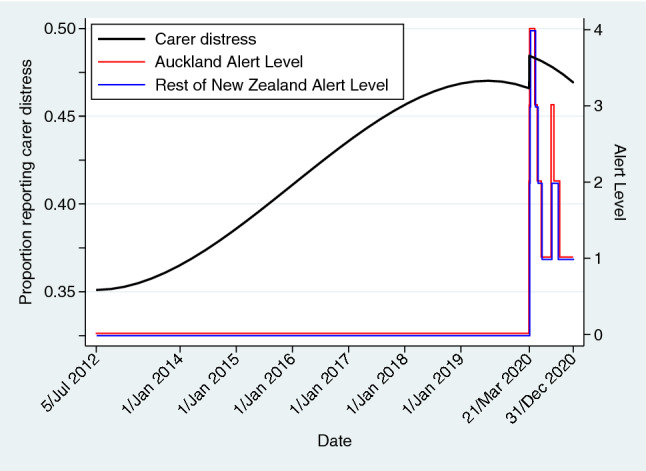
The best fitting fractional polynomial model of recorded carer distress over time for first interRAI-HC assessments, together with the COVID-19 Alert Level changes for Auckland and the rest of New Zealand.

As part of the New Zealand Government’s response to the COVID-19 pandemic, on the 21 March 2020 it introduced a 4-tiered Alert Level system aimed initially at eliminating the virus^35^. Depending on community case numbers, scientific knowledge, and control measures effectiveness, Alert Levels could increase or decrease, and be different between various geographical regions. Four days after this announcement, New Zealand, as a nation, moved to Alert Level 4 and into lockdown. All travel (including local), gatherings, workplaces, and services were heavily restricted^35^, and individual and societal uncertainty was high. While the initial achievement of COVID-19 elimination was regarded as being successful^36^, many older adults continued to self-isolate and felt vulnerable even as the Alert Levels decreased. New Zealand’s Alert Level history over the study duration is graphically displayed in Fig. 1^35^.

### Sociodemographic and selected health measures

Older adult sex response options were: male, female, unknown, and indeterminate; responses to the last two options were set to missing. Age was derived from the difference between date of birth and date of interview. Using the Ministry of Health’s level one ethnicity coding protocols, older adults with multiple identifications were assigned a single ethnicity via the prioritization hierarchy: (i) Māori; (ii) Pacific; (iii) Asian; and, (iv) European/other. Marital status was elicited with six response options: never married; married/civil union/de facto; widowed; separated; divorced; other. For these analyses, separated and divorced categories were combined. Living arrangements at the time of the assessment had response options: alone, with spouse/partner only, with spouse/partner and other(s), with child (not spouse/partner), with parent(s) or guardian(s), with sibling(s), with relatives, and with non-relative(s). Participants can report up to two informal carers. Informal caregiving is usually done by one main caregiver, who might be supported by other family members and/or by additional formal care providers^29^. The first nominated person gives the main carer, and defined as being their child or child-in-law, spouse, partner/significant other, parent/guardian, sibling, other relatives of family (whanau), friend, or neighbour. These were collapsed into four categories: child or child-in-law, spouse/partner, relatives, and no-relative groups. However, the gender of the carer(s) was neither elicited nor recorded. Within New Zealand, 20 District Health Boards (DHBs) are responsible for providing or funding the provision of health services within their district. Geographically defined, DHBs have different governance groups and policies, while their constituents have varying sociodemographic profiles, health needs and conditions.

Selected health measures were based on their known association between dependence-level of those being cared for and caregiver burden^10,14^. The Method for Assigning Priority Levels (MAPLe) score, an algorithm for prioritizing access to community and facility-based services for home care clients, was utilised and employed^37^. This algorithm yielded five different groups ranging from low to very high risk. Participation in activities of daily living (ADL) was assessed using the ADL hierarchy scale^38^, which combines four items (personal hygiene, toilet transfer, locomotion, and eating) to create a seven-category score: independence; supervision; limited; extensive; maximal; dependent; and, total dependence. Finally, cognitive impairment was assessed using the Cognitive Performance Scale (CPS)^39^, which combined five items (daily decision making, memory recall ability, periodic disordered thinking or awareness, acute change in mental status, change in decision making) to create a seven-category score: intact; broad-line intact; mild; moderate; moderate severe; severe; and, very severe.

### Procedure

A detailed account of the interRAI-HC assessment instrument and procedure within New Zealand has been described previously^31^. In brief, the interRAI-HC instrument is employed for the conduct of all community care assessments of older people needing publically funded long-term community services or aged residential care admission. After referral from a health practitioner, these adults have their needs assessed by trained interRAI assessors. Typically, assessments are home-based, and are primarily used to develop individualized care-plans according to a standardized protocol. All assessed adults are explicitly asked if they consent to their de-identified interRAI-HC information being used for planning and research purposes. Approximately 93% provide this consent^31^. Only consented data are made available for research.

### Statistical analysis

Reporting of analyses were informed by the REporting of studies Conducted using Observational Routinely collected health Data (RECORD) guidelines^40^. Assessment and participant flow eligibility numbers were initially reported, followed by descriptive statistics of the participant characteristics at the first assessment together with assessment frequency and inter-assessment periodicity. Crude frequencies of carer distress were then reported, followed by an investigation of the pattern of carer distress over time using fractional polynomial regression models^41^. These models utilised only the first assessment information, included a COVID-19 Alert Level indication (0 when Alert Level = 0; 1 otherwise), and were constrained to the study period (5 July 2012 and 31 December 2020, inclusive). Consistent with the recommendations of Royston and Sauerbrei^41^, degree-2 fractional polynomial powers of time were considered from the set (− 2; − 1; − 0.5; 0; 0.5; 1; 2; 3), where time was scaled from 4 July 2012 (with value 0) to 31 December 2020 (with value 1). The best model was selected by minimising the deviance statistic, and the χ^2^ test used to investigate deviance differences between models.

Once the time and step patterns were identified, these were employed in a multivariable multilevel mixed-effects model which utilised the full eligible research dataset. This model treated participants as random effects and assessments as being nested within. Conventionally employed logistic regression models produce odds ratios that are biased and inflated estimates of relative risks (RRs) when the outcome of interest is not rare, yet are commonly used and interpreted as such^42^. Because carer distress was relatively common, a modified Poisson regression approach (with log-link function and robust variance estimators) was used to estimate RRs directly^42^. Multilevel mixed-effects modified Poisson regression models were thus employed for this multivariable analyses. Rather than employing bivariable analyses to screen risk factors, in the spirit of Sun and colleagues^43^, all candidate variables were included in the adjusted model regardless of their statistical significance. RRs and associated 95% confidence intervals (CIs) were reported, and Wald’s type III χ^2^ statistic used to determine the significance of variables within this regression models. Because the sample size was so large (with n ≥ 25,000), the Hosmer–Lemeshow goodness-of-fit test is not recommended and was not calculated^44^. Instead the model’s predictive accuracy was assessed by an area under the receiver operating characteristic curve (AUC) analysis^45^. Adopting the recommendations of Hosmer and Lemeshow, an AUC of 0.5 suggests no discrimination, 0.7–0.8 is considered acceptable, 0.8–0.9 is considered excellent and more than 0.9 is considered outstanding^46^. Population attributional fractions were then ascertained, adapting the method introduced by Greenland and Drescher^47^. Sensitivity analyses were conducted, using chained equations multiple imputation (M = 50) methods for all variables within the adjusted model. All analyses and graphics were performed using Stata SE version 17.0 (StataCorp, College Station, TX, USA), and α = 0.05 defined statistical significance.

### Ethics

Clearance for this secondary analysis of routinely collected de-identified data study was approved by the Ministry of Health’s Health and Disability Ethics Committees (14/STH/140/AM07). All methods were performed in accordance with that Ethics Committee’s relevant guidelines and regulations. The study only included those participants who, at the time of their interRAI-HC assessment, provided written and informed consent to their data being used for planning and research purposes. The Ministry of Health does not release data to researchers for those who do not provide this consent.

## Results

### Participants and their characteristics

Overall, the extracted interRAI-HC dataset contained 278,268 assessments from 169,703 participants. However, 14,023 assessments were undertaken on people aged < 65 years, 18,425 assessments were with individuals’ whose residential status at the time of assessment was not their private home, apartment or rented room, in 12,764 assessments participants had no informal carer, and in 1784 assessments the repeated assessment date was within 30 days, leaving 231,277 eligible assessments from 144,358 older adults. Some 86,318 (59.8%) participants had one assessment, 38,239 (26.5%) had two, 13,682 (9.5%) had three, 4093 (2.8%) had four, 1349 (0.9%) had five, 472 (0.3%) had six, 167 (0.1%) had seven, 31 (0.02%) had eight, 6 (< 0.01%) had nine, and 1 (< 0.01%) participant had ten separate assessments. The median time between assessments was 14 months (Q_1_ = 9, Q_3_ = 24 months), with the largest assessment interval being 8.2 years. The participants’ average age at their first assessment was 82.0 years (range: 65–107 years), and their demographic profiles are presented in Table 1. Females predominated, and most were aged between 75 and 94 years, were of European/other ethnic identification, and lived with their spouse partner or alone.

**Table 1:** Distribution of participants’ sociodemographic measures at their first interRAI-HC assessment.

	n	(%)
**Sex**^**a**^		
Female	85,676	(59.4)
Male	58,582	(40.6)
**Age group (years)**		
65–74	26,314	(18.2)
75–84	58,831	(40.8)
85–94	54,369	(37.7)
≥ 95	4844	(3.4)
**Ethnic identification**		
European/other	126,208	(87.4)
Māori	8815	(6.1)
Pacific	4778	(3.3)
Asian	4557	(3.2)
**Marital status**^**b**^		
Married/de facto	60,300	(41.9)
Widowed	65,192	(45.3)
Divorced/separated	11,080	(7.7)
Never married	5984	(4.2)
Other	1513	(1.1)
**Living arrangements**		
Spouse/partner only	49,098	(34.0)
Spouse/partner and other(s)	5927	(4.1)
Alone	66,870	(46.3)
Child, no spouse/partner	16,825	(11.7)
Other relative(s)	3868	(2.7)
Non-relative(s)	1770	(1.2)

^a^Sex was unknown for 98 and indeterminate for 2 participants.

^b^289 values were missing.

### Carers and carer distress

At the first assessment, one primary carer and no secondary carer was identified for 45,708 (31.7%) participants, while both primary and secondary caregivers were recorded for the remaining 98,650 (68.3%) participants. At these assessments, the relationship of the primary caregiver to the participant was child or child-in-law (73,516; 50.9%), spouse or partner (48,192; 33.4%), other relative (11,141; 7.7%), and non-relative (11,509; 8.0%). For the secondary caregivers, their relationship was child or child-in-law (70,744; 71.7%), spouse or partner (1,808; 1.8%), other relatives (13,657; 13.8%), and non-relative (12,441; 12.6%). The most common carer relationship figurations at these first assessments were child or child-in-law as both primary and secondary (39,461; 27.3%), spouse or partner as primary and child or child-in-law as secondary (27,411; 19.0%), child or child-in-law as primary with no secondary (20,182, 14.0%), and spouse or partner as primary with no secondary carer (16,457; 11.4%).

Valid, non-missing responses to the three variables comprising carer distress were available for 143,713 (99.6%) first assessments and 230,581 (99.7%) of all eligible assessments. Overall, carer distress was indicated in 59,942 (41.7%) first assessments and 101,819 (44.2%) of all assessments. The best fitting fractional polynomial model of first assessments, which also included the COVID-19 step function, had powers *time*^2^ and *time*^3^, and estimated regression coefficients:

$${\text{proportion with carer distress }} = \, 0.{351}0 \, + \, \left( {0.{5311} \times time^{{2}} } \right) \, {-} \, \left( {0.{4314} \times time^{{3}} } \right) \, + \, \left( {0.0{186} \times {\text{COVID}}} \right)$$

where *time* was scaled, starting from 4 July 2012 (with value 0) to 31 December 2020 (with value 1), and COVID = 0 for the period before 21 March 2020 (i.e., 0 < *time* < 0.9081) and COVID = 1 for the period thereafter (i.e., 0.9081 ≤ *time* ≤ 1). In this model the time components were statistically significant (both p < 0.001), as was the COVID step function (95% CI 0.0018, 0.0354; p = 0.03). This regression curve is superimposed on the COVID-19 Alert Level changes for Auckland and the rest of New Zealand over the study period depicted in Fig. 1. Substituting time values into this equation yielded carer distress prevalence estimates, which increased from 35.1% (95% CI 34.6%, 35.6%) on 5 July 2012 to a peak of 48.5% (95% CI 47.4%, 49.5%) on 21 March 2020. By the end of the study period, 31 December 2020, the estimated prevalence of carer distress was 46.9% (45.9%, 47.9%).

### Multivariable multilevel mixed-effects analysis

Adopting the identified *time*^2^ and *time*^3^ powers, a multilevel mixed-effects Poisson model was conducted on the full research dataset, treating participants as random effects and assessments nested within. Complete information for the considered variables was available for 230,074 assessments from 143,766 participants. Table 2 gives the distribution of carer distress indications for participant characteristic and potentially confounding variables at the first assessment, together with estimated RRs and associated 95% CIs estimated from the multivariable multilevel mixed-effects Poisson model. Again, these time components were statistically significant (both p < 0.001), as was the COVID step function with RR = 1.06 (95% CI 1.04, 1.09; p < 0.001).

**Table 2:** Distribution of carer distress indications for participants’ sociodemographic and selected health measures at the first assessment, together with estimated relative risks (RRs) and associated 95% confidence intervals (CIs) estimated from the crude and adjusted multilevel mixed-effects Poisson models.

	First assessment			All assessments			
	Total	Carer distress		Crude analysis^a^		Adjusted analysis^b^	
		n	(%)	IRR	(95% CI)	IRR	(95% CI)
*time*^2^	–	–		3.76	(3.31, 4.26)	3.64	(3.25, 4.09)
*time*^3^	–	–		0.34	(0.30, 0.39)	0.33	(0.29, 0.38)
**COVID indication**							
Pre-21 March 2020	132,978	54,821	(41.2)	1	(reference)	1	(reference)
On or post-21 March 2020	10,735	5121	(47.7)	1.06	(1.03, 1.09)	1.06	(1.04, 1.09)
*Sex*							
Female	85,281	31,904	(37.4)	1	(reference)	1	(reference)
Male	58,332	27,992	(48.0)	1.27	(1.26, 1.28)	1.05	(1.04, 1.06)
**Age group (years)**							
65–74	26,186	11,482	(43.8)	1	(reference)	1	(reference)
75–84	58,580	25,067	(42.8)	1.00	(0.98, 1.01)	0.99	(0.98, 1.00)
85–94	54,117	21,538	(39.8)	0.93	(0.92, 0.95)	0.97	(0.96, 0.99)
≥ 95	4830	1855	(38.4)	0.89	(0.86, 0.92)	0.97	(0.94, 1.00)
**Ethnic identification**							
European/other	125,642	52,386	(41.7)	1	(reference)	1	(reference)
Māori	8786	3,502	(39.9)	0.96	(0.94, 0.98)	0.92	(0.90, 0.95)
Pacific	4751	1770	(37.3)	0.88	(0.85, 0.91)	0.87	(0.84, 0.90)
Asian	4534	2284	(50.4)	1.09	(1.06, 1.13)	1.05	(1.02, 1.08)
**Marital status**							
Married/de facto	60,025	31,755	(52.9)	1	(reference)	1	(reference)
Widowed	64,900	21,555	(33.2)	0.64	(0.63, 0.64)	0.91	(0.89, 0.93)
Divorced/separated	11,039	3955	(35.8)	0.66	(0.64, 0.67)	0.96	(0.93, 0.99)
Never married	5948	1975	(33.2)	0.60	(0.58, 0.62)	0.94	(0.90, 0.98)
Other	1512	567	(37.5)	0.67	(0.63, 0.71)	0.94	(0.89, 1.00)
**Living arrangements**							
Spouse/partner only	48,879	26,764	(54.8)	1	(reference)	1	(reference)
Spouse/partner & other(s)	5897	3070	(52.1)	0.94	(0.92, 0.97)	0.99	(0.97, 1.01)
Alone	66,587	20,158	(30.3)	0.55	(0.54, 0.56)	0.81	(0.79, 0.83)
Child, no spouse/partner	16,737	7536	(45.0)	0.81	(0.80, 0.83)	1.05	(1.02, 1.08)
Other relative(s)	3852	1678	(43.6)	0.77	(0.74, 0.79)	1.05	(1.01, 1.09)
Non-relative(s)	1761	736	(41.8)	0.75	(0.72, 0.79)	1.09	(1.04, 1.15)
**Primary carer relationship**							
Child or child-in-law	73,181	26,241	(35.9)	1	(reference)	1	(reference)
Spouse/partner	47,983	27,140	(56.6)	1.56	(1.55, 1.58)	1.15	(1.12, 1.17)
Other relative	11,086	3784	(34.1)	0.92	(0.89, 0.94)	0.94	(0.91, 0.96)
Non-relative	11,463	2777	(24.2)	0.67	(0.65, 0.69)	0.76	(0.74, 0.78)
**Number of carers**							
One	45,462	19,194	(42.2)	1.02	(1.01, 1.03)	1.04	(1.03, 1.05)
Two	98,251	40,748	(41.5)	1	(reference)	1	(reference)
**MAPLe categories**							
Low priority	26,079	4650	(17.8)	1	(reference)	1	(reference)
Mild priority	8815	2171	(24.6)	1.41	(1.36, 1.47)	1.35	(1.30, 1.40)
Moderate priority	30,220	11,993	(39.7)	2.43	(2.36, 2.50)	1.76	(1.71, 1.81)
High priority	53,239	25,631	(48.1)	2.92	(2.84, 3.00)	1.94	(1.88, 1.99)
Very high priority	25,327	15,478	(61.1)	3.93	(3.83, 4.04)	2.27	(2.20, 2.34)
**ADL hierarchy**							
Independent	84,009	27,456	(32.7)	1	(reference)	1	(reference)
Supervision required	20,280	10,840	(53.5)	1.69	(1.67, 1.71)	1.26	(1.24, 1.28)
Limited assistance	15,964	8057	(50.5)	1.62	(1.60, 1.64)	1.22	(1.20, 1.24)
Extensive assistance	9740	5532	(56.8)	1.82	(1.79, 1.84)	1.27	(1.25, 1.29)
Maximal assistance	6490	3837	(59.1)	1.86	(1.82, 1.89)	1.30	(1.28, 1.33)
Very dependent	6302	3661	(58.1)	1.83	(1.79, 1.86)	1.29	(1.27, 1.32)
Total dependence	924	558	(60.4)	1.85	(1.77, 1.94)	1.31	(1.25, 1.38)
**Cognitive performance scale (CPS)**							
Intact	44,329	11,655	(26.3)	1	(reference)	1	(reference)
Borderline intact	26,234	9231	(35.2)	1.37	(1.34, 1.39)	1.18	(1.16, 1.21)
Mild impairment	50,168	23,746	(47.3)	1.84	(1.81, 1.87)	1.23	(1.21, 1.26)
Moderate impairment	15,851	10,402	(65.6)	2.56	(2.51, 2.60)	1.40	(1.37, 1.43)
Mod./severe impairment	1511	1059	(70.1)	2.71	(2.64, 2.79)	1.36	(1.32, 1.40)
Severe impairment	4907	3409	(69.5)	2.76	(2.70, 2.81)	1.35	(1.32, 1.38)
Very severe impairment	711	439	(61.7)	2.36	(2.25, 2.47)	1.17	(1.10, 1.23)
**District health board (DHB)**							
Northland	5487	2584	(47.1)	1.42	(1.37, 1.47)	1.37	(1.32, 1.41)
Waitemata	9822	6484	(66.0)	1.86	(1.81, 1.91)	1.62	(1.58, 1.66)
Auckland	9862	3498	(35.5)	1	(reference)	1	(reference)
Counties Manukau	12,875	4300	(33.4)	0.99	(0.96, 1.02)	1.05	(1.01, 1.08)
Bay of Plenty	10,688	4405	(41.2)	1.20	(1.16, 1.24)	1.25	(1.22, 1.29)
Waikato	13,392	5889	(44.0)	1.31	(1.27, 1.35)	1.38	(1.34, 1.42)
Lakes	3520	1451	(41.2)	1.19	(1.14, 1.25)	1.35	(1.30, 1.41)
Tairawhiti	1531	502	(32.8)	0.99	(0.93, 1.06)	1.10	(1.04, 1.17)
Taranaki	5199	1606	(30.9)	0.97	(0.93, 1.01)	1.11	(1.07, 1.16)
Whanganui	2716	1010	(37.2)	1.18	(1.13, 1.24)	1.27	(1.22, 1.32)
Hawke's Bay	7430	3176	(42.7)	1.32	(1.28, 1.37)	1.46	(1.41, 1.50)
MidCentral	6445	2475	(38.4)	1.15	(1.11, 1.19)	1.16	(1.12, 1.20)
Capital and coast	8931	2887	(32.3)	0.92	(0.89, 0.96)	1.03	(1.00, 1.07)
Hutt Valley	4585	1934	(42.2)	1.17	(1.13, 1.22)	1.20	(1.16, 1.24)
Wairarapa	2254	691	(30.7)	1.00	(0.94, 1.05)	1.11	(1.06, 1.17)
Nelson Marlborough	6380	1809	(28.4)	0.89	(0.86, 0.93)	0.97	(0.94, 1.01)
West coast	1243	286	(23.0)	0.74	(0.68, 0.81)	0.84	(0.77, 0.91)
Canterbury	15,518	6680	(43.0)	1.29	(1.25, 1.33)	1.41	(1.37, 1.45)
South Canterbury	3135	1144	(36.5)	1.07	(1.03, 1.12)	1.25	(1.20, 1.30)
Southern	12,700	7131	(56.1)	1.73	(1.69, 1.78)	1.84	(1.79, 1.89)

^a^Adjusted for *time*^2^, *time*^3^, and COVID indication.

^b^Adjusted for all variables contained within Table 2.

All the participant characteristic and potentially confounding variables were significantly related to carer distress (all p < 0.001, except age group p = 0.002), with the MAPLe score (χ^2^ = 3,185, d.f. = 4), DHB regions (χ^2^ = 6,454, d.f. = 19), and ADL scores (χ^2^ = 1,797, d.f. = 6) explaining a relatively higher proportion of the variance. The population attributional fraction of carer distress experience associated with the COVID-19 period was estimated as 0.56% (95% CI 0.35%, 0.77%), implying that approximately one in every 180 instances of carer distress could be attributed to the COVID-19 Alert Level introduction (and restrictions). With predictions based on fixed effects and posterior means of the random effects, the AUC = 0.757 (95% CI 0.755, 0.759), a value which is considered acceptable suggesting the model had adequate fit.

### Sensitivity analyses

After undertaking chained equations MI for missing data (M = 50), and repeating the multivariable multilevel mixed-effects modified Poisson model, the resulting estimates were strikingly similar to those derived from the complete case analyses; see Supplementary Table S1. In terms of absolute difference in estimated adjusted RRs, the maximum change between complete case and MI estimates was only ± 0.01 for all considered variables; a negligible difference. The mean estimated ROC area for these M = 50 multiple imputations was 0.757 (95% CI 0.755, 0.759); identical to the complete case estimate (at 3-decimal points) and a level that continues to represent acceptable predictive accuracy.

## Discussion

Using a large, national, population-based study of older adults with complex needs, the prevalence of distress recorded for their informal carer was high and has rapidly increased in recent years. At its measured peak, almost one from every two participants had distress recorded for their informal caregiver at their first assessment, compared to just over one in every three participants at the beginning of the study in 2012. The underlying reasons for this are likely to be multifactorial and multilevel—underpinned by individual, community and societal change. One factor may be older adults’ decompression of morbidity, whereby gains in life expectancy are not matched by gains in years of independent function^48^. As duration of caring is among the most important predictors of caregiver burden^10,14^, decompression of morbidity is likely to evoke sustained increased distress. Another important influence might result from the intensifying pressures on the ‘sandwich generation’—those supporting both children and parents^49,50^. This generation is also suffering from increasing financial stress^50^. Women make up the overwhelming majority of those in unpaid caring roles, and it remains a societal expectation that they will undertake essential unpaid work which perpetuates gender financial inequality^51^. Despite increases in female employment rates (which add additional time pressures)^51^, the rampant housing market in New Zealand has seen house and rent price appreciation which persistently outstrip income growth over the study period^52^. Thus it is perhaps unsurprising that increasingly time and resource poor women who are providing informal care to their older parent(s) or parent(s)-in-law with decompressed morbidity are becoming more stressed.

In terms of the COVID-19 effect of increasing caregiver distress for those caring for older adults with complex needs, a statistically significant but clinically negligible increase was observed with the introduction of the Government’s Alert Levels and the swift move into lockdown and self-isolation. Moreover, the initial step function increase appeared to dampen relatively quickly—returning to the plateauing pre-COVID-19 levels of distress. Based on the multivariable multilevel mixed effects analysis, only approximately one in every 180 carer distress reports could be attributed to the COVID-19 Alert Level introduction and change. So while there is a suggestion that COVID-19 has increased the strain on both time and finances for the ‘sandwich generation’^50^, and that there is a decline in carers mental health as the pandemic progresses^29,53^, the additional measured increase in carer distress within New Zealand did not appear to be sustained. Indeed, it paled by comparison to other underlying factors—at least to the end of 2020. In a recently published scoping review, it was identified that the pandemic exacerbated pre-existing issues for carers (namely: psychological well-being, personal health and well-being, social support, disruption to routines, financial concerns, inability to access healthcare and medications) as well as causing new concerns^53^. It may be that these new concerns will have a greater influence on New Zealand’s carers as COVID-19 transmission becomes widespread within their community^20,35^.

Within the multivariable model, all considered participant characteristic and potentially confounding variables were significantly related to carer distress, although the estimated effect sizes were relatively small and inconsequential for age group and marital status. Given that dependence-level of those being cared for is an important predictor of caregiver burden^10,14^, it was unsurprising that increasing MAPLe and ADL scores explained a relatively higher proportion of the model’s variance, and that moderate to severe cognitive impairment was also associated with an approximately 40% increased risk of recorded carer distress. Noteworthy was that spousal or partner primary caregivers of male participants had substantially increased risk of distress relative to their peers. This reflects a number of complex intertwined individual and societal factors associated with the gendered nature of caregiving^51,54^. Heterosexual men are more likely to have a spouse/partner able to provide care, because of women’s longer life-expectancy, their typically younger age, and through society’s expectations and norms. Consequently, men utilise and receive less formal service support; whereas women in advanced age are more likely to live alone and rely upon this outside support^54,55^. Spousal carers are also likely to be juggling their own health issues as comorbidities and functional decline increase with time. It is also worth noting that there was a strong DHB effect. Compared to Auckland, which caters for the country’s largest and most diverse population group, risk of carer distress ranged from 0.84 (West Coast) to 1.84 (Southern). The precise mechanism for these variations is speculative, but likely a combination of the different access to and provisions of health services, together with the considerably different socio-demographic profiles and population needs.

Ethnic identification was also strongly associated with carer distress in the adjusted analyses. Compared to the dominant European/other group, participants who were Māori or Pacific had carers with less risk of distress, while Asian participants had slightly higher risk. In New Zealand, Māori are the indigenous people who first discovered and occupied the country. Māori today share a common ancestry with modern-day Polynesians, but are considered a distinct ethnic group from their more recent Pacific arrivals. Both Māori and Pacific people have shared traditional values of collective interdependence and communitarianism—working cooperatively toward shared goals; reciprocity; respect (especially toward elders, parents, women, and people in positions of authority); and spirituality—accrediting life events to a higher power. While acculturation and assimilation exert continuous cultural pressures and these values are dynamic and evolve over time, they continue to prevail for many. Both Māori and Pacific people are more likely to live in multigenerational households, which perversely means that with more informal care available within their homes, they are less likely to qualify for formal support^55^. And, indeed, patterns of informal caregiving have been shown to be significantly higher for older Māori adults compared to their non-Māori counterparts^54^. Yet, despite providing more informal care, carer distress for both Māori and Pacific participants was less. Because of these traditional values, it has been argued that informal caregiving for Māori (and Pacific) can be perceived as a cultural responsibility and is supported by wider whānau (extended family) connections^54^. Care is thus a shared task, supported by others, and it confers respect and alignment with their interdependence and communitarianism values; all factors which are likely to reduce caregiving distress. Although, these different cultural expectations and values may also result in differential under-reporting, masking the underlying extent of this carer distress in these communities.

### Strengths and limitations

While having notable strengths, such as the large, national, contemporary, prospectively collected repeated measured dataset using a standardised validated instrument which captures a suite of variables with very little missing data, together with the careful data analysis and reporting of findings according to best practice guidelines, this study also has limitations. Arguably, the carer distress primary variable, the interRAI-HC selection mechanism, and unmeasurable confounding potentially represent the largest threats to the study’s validity. Although recorded carer distress, as defined in this study, has good face validity and been employed elsewhere^11,32–34^, it has not been subject to rigorous psychometric testing. Results from systematic reviews and meta-analyses identified that there was substantial variation in the types of measures used^11,13^, and that future research should consolidate measures to enable more precise identification^11^. Also the gender of the carer is not elicited in the interRAI-HC. Given the gendered nature and distribution of care giving, and the notable gender differences observed within this study, this omission blunts the gender-specific nuance and understanding of carer distress. Another limitation is that, due to COVID-19, changes were made in how overdue repeat interRAI-HC assessments were collected within New Zealand. Pre-COVID-19, these were invariably conducted in person, while after the Alert Levels were instigated, phone assessments were also used and some were conducted by senior nursing students. This may have impacted on how carer distress was detected; although first assessments predominated the study dataset—and only these assessments were used in the initial fractional polynomial regression modelling. Within New Zealand, the interRAI-HC assessment is required for people seeking publicly funded support services. A small percentage chose to use private funds for these services; a group which is likely to be the relatively affluent and who have health benefits associated from these resources. While self-referral is possible, older adults invariably present to a health practitioner for an interRAI-HC assessment referral. However, ethnic inequities in access to primary health care remains in New Zealand, with Māori and Pacific people less likely than non-Māori/non-Pacific people to access care, and Māori and Pacific people carry a disproportionate burden of disease^31^. So while the study cohort is large and ethnically diverse, it is not completely representative of New Zealand adults with complex needs aged ≥ 65 years. Finally, though the adjusted model has adequate fit, with AUC = 0.757 (95% CI 0.755, 0.759), there likely remains unmeasured confounders. Such confounders can result in substantial bias in the estimated exposure-outcome adjusted RR, particularly if they are uncorrelated with the measured explanatory variables^56^. Study replication using different suites of variables is needed to understand their effect.

## Conclusions

Nationally, New Zealand has witnessed a dramatic increase in carer distress among many thousands of older adults receiving interRAI-HC assessments. If informal caregiving in home settings is to remain a cornerstone of New Zealand’s ageing in place policies, then this hitherto unmeasured widespread level of carer distress within this country represents a significant and potentially catastrophic risk. Recognition of this risk, and developing and implementing apposite policies and services providing efficacious strategies to support caregivers deserves specific attention.

## Supplementary Information

Supplementary Information.

## Data Availability

The data that support the findings of this study are available from New Zealand’s Ministry of Health but restrictions apply to the availability of these data, which were used under license for the current study, and so are not publicly available. Data are however available from the corresponding author upon reasonable request and with permission of interRAI New Zealand (see: https://www.interrai.co.nz/data-research-and-reporting/requesting-interrai-data/).
