# Supplementary material for: Carer distress among community living older adults with complex needs in the pre- and post-COVID-19 era: a national population study
Source: Sci Rep. 2022 Nov 16;12:19697. doi: 10.1038/s41598-022-24073-0 (PMC9668208; doi:10.1038/s41598-022-24073-0)
Supplement: Supplementary file 1 — Supplementary Information. [file 41598_2022_24073_MOESM1_ESM.docx]

**Supplementary materials for:**

**Carer distress among community living older adults with complex needs in the pre- and post-COVID-19 era: a national population study**

**Table S1**. Distribution of carer distress indications for participant characteristic and potentially confounding variables at the first assessment, together with estimated relative risks (RRs) and associated 95% confidence intervals (CIs) estimated from the adjusted multilevel mixed-effects Poisson model, and the multiple imputed (MI) adjusted multilevel Poisson model.

|  |  | **First assessment** | | |  | | **All assessments** | | | |
| --- | --- | --- | --- | --- | --- | --- | --- | --- | --- | --- |
|  |  |  | Carer distress | | | Adjusted analysis^a^ | | | MI adjusted analysis^a^ | |
|  |  | Total | n | (%) | | IRR | | (95% CI) | IRR | (95% CI) |
| *time*^2^ | | - | - |  | | 3.64 | | (3.25, 4.09) | 3.65 | (3.26, 4.10) |
| *time*^3^ | | - | - |  | | 0.33 | | (0.29, 0.38) | 0.33 | (0.29, 0.37) |
| *COVID indication* | |  |  |  | |  | |  |  |  |
|  | Pre-21 March 2020 | 132,978 | 54,821 | (41.2) | | 1 | | (reference) | 1 | (reference) |
|  | On or post-21 March 2020 | 10,735 | 5,121 | (47.7) | | 1.06 | | (1.04, 1.09) | 1.06 | (1.04, 1.09) |
| *Sex* | |  |  |  | |  | |  |  |  |
|  | Female | 85,281 | 31,904 | (37.4) | | 1 | | (reference) | 1 | (reference) |
|  | Male | 58,332 | 27,992 | (48.0) | | 1.05 | | (1.04, 1.06) | 1.05 | (1.04, 1.06) |
| *Age group (years)* | |  |  |  | |  | |  |  |  |
|  | 65-74 | 26,186 | 11,482 | (43.8) | | 1 | | (reference) | 1 | (reference) |
|  | 75-84 | 58,580 | 25,067 | (42.8) | | 0.99 | | (0.98, 1.00) | 0.99 | (0.98, 1.00) |
|  | 85-94 | 54,117 | 21,538 | (39.8) | | 0.97 | | (0.96, 0.99) | 0.97 | (0.96, 0.99) |
|  | ≥95 | 4,830 | 1,855 | (38.4) | | 0.97 | | (0.94, 1.00) | 0.97 | (0.94, 1.00) |
| *Ethnic identification* | |  |  |  | |  | |  |  |  |
|  | European/other | 125,642 | 52,386 | (41.7) | | 1 | | (reference) | 1 | (reference) |
|  | Māori | 8,786 | 3,502 | (39.9) | | 0.92 | | (0.90, 0.95) | 0.92 | (0.90, 0.94) |
|  | Pacific | 4,751 | 1,770 | (37.3) | | 0.87 | | (0.84, 0.90) | 0.87 | (0.84, 0.90) |
|  | Asian | 4,534 | 2,284 | (50.4) | | 1.05 | | (1.02, 1.08) | 1.05 | (1.02, 1.08) |
| *Marital status* | |  |  |  | |  | |  |  |  |
|  | Married/de facto | 60,025 | 31,755 | (52.9) | | 1 | | (reference) | 1 | (reference) |
|  | Widowed | 64,900 | 21,555 | (33.2) | | 0.91 | | (0.89, 0.93) | 0.91 | (0.89, 0.93) |
|  | Divorced/separated | 11,039 | 3,955 | (35.8) | | 0.96 | | (0.93, 0.99) | 0.96 | (0.93, 0.99) |
|  | Never married | 5,948 | 1,975 | (33.2) | | 0.94 | | (0.90, 0.98) | 0.94 | (0.90, 0.98) |
|  | Other | 1,512 | 567 | (37.5) | | 0.94 | | (0.89, 1.00) | 0.94 | (0.89, 1.00) |
| *Living arrangements* | |  |  |  | |  | |  |  |  |
|  | Spouse/partner only | 48,879 | 26,764 | (54.8) | | 1 | | (reference) | 1 | (reference) |
|  | Spouse/partner and other(s) | 5,897 | 3,070 | (52.1) | | 0.99 | | (0.97, 1.01) | 0.99 | (0.97, 1.01) |
|  | Alone | 66,587 | 20,158 | (30.3) | | 0.81 | | (0.79, 0.83) | 0.81 | (0.79, 0.83) |
|  | Child, no spouse/partner | 16,737 | 7,536 | (45.0) | | 1.05 | | (1.02, 1.08) | 1.05 | (1.02, 1.08) |
|  | Other relative(s) | 3,852 | 1,678 | (43.6) | | 1.05 | | (1.01, 1.09) | 1.05 | (1.01, 1.10) |
|  | Non-relative(s) | 1,761 | 736 | (41.8) | | 1.09 | | (1.04, 1.15) | 1.09 | (1.04, 1.15) |
| *Primary carer relationship* | |  |  |  | |  | |  |  |  |
|  | Child or child-in-law | 73,181 | 26,241 | (35.9) | | 1 | | (reference) | 1 | (reference) |
|  | Spouse/partner | 47,983 | 27,140 | (56.6) | | 1.15 | | (1.12, 1.17) | 1.15 | (1.12, 1.17) |
|  | Other relative | 11,086 | 3,784 | (34.1) | | 0.94 | | (0.91, 0.96) | 0.93 | (0.91, 0.96) |
|  | Non-relative | 11,463 | 2,777 | (24.2) | | 0.76 | | (0.74, 0.78) | 0.76 | (0.74, 0.78) |
| *Number of carers* | |  |  |  | |  | |  |  |  |
|  | One | 45,462 | 19,194 | (42.2) | | 1.04 | | (1.03, 1.05) | 1.04 | (1.03, 1.05) |
|  | Two | 98,251 | 40,748 | (41.5) | | 1 | | (reference) | 1 | (reference) |
| *MAPLe categories* | |  |  |  | |  | |  |  |  |
|  | Low priority | 26,079 | 4,650 | (17.8) | | 1 | | (reference) | 1 | (reference) |
|  | Mild priority | 8,815 | 2,171 | (24.6) | | 1.35 | | (1.30, 1.40) | 1.35 | (1.30, 1.41) |
|  | Moderate priority | 30,220 | 11,993 | (39.7) | | 1.76 | | (1.71, 1.81) | 1.76 | (1.71, 1.81) |
|  | High priority | 53,239 | 25,631 | (48.1) | | 1.94 | | (1.88, 1.99) | 1.94 | (1.89, 2.00) |
|  | Very high priority | 25,327 | 15,478 | (61.1) | | 2.27 | | (2.20, 2.34) | 2.27 | (2.21, 2.34) |
| *ADL hierarchy* | |  |  |  | |  | |  |  |  |
|  | Independent | 84,009 | 27,456 | (32.7) | | 1 | | (reference) | 1 | (reference) |
|  | Supervision required | 20,280 | 10,840 | (53.5) | | 1.26 | | (1.24, 1.28) | 1.26 | (1.24, 1.28) |
|  | Limited assistance | 15,964 | 8,057 | (50.5) | | 1.22 | | (1.20, 1.24) | 1.22 | (1.20, 1.24) |
|  | Extensive assistance | 9,740 | 5,532 | (56.8) | | 1.27 | | (1.25, 1.29) | 1.27 | (1.25, 1.29) |
|  | Maximal assistance | 6,490 | 3,837 | (59.1) | | 1.30 | | (1.28, 1.33) | 1.30 | (1.28, 1.33) |
|  | Very dependent | 6,302 | 3,661 | (58.1) | | 1.29 | | (1.27, 1.32) | 1.29 | (1.27, 1.32) |
|  | Total dependence | 924 | 558 | (60.4) | | 1.31 | | (1.25, 1.38) | 1.31 | (1.25, 1.38) |
| *Cognitive performance scale (CPS)* | | |  |  | |  | |  |  |  |
|  | Intact | 44,329 | 11,655 | (26.3) | | 1 | | (reference) | 1 | (reference) |
|  | Borderline intact | 26,234 | 9,231 | (35.2) | | 1.18 | | (1.16, 1.21) | 1.18 | (1.16, 1.21) |
|  | Mild impairment | 50,168 | 23,746 | (47.3) | | 1.23 | | (1.21, 1.26) | 1.23 | (1.21, 1.26) |
|  | Moderate impairment | 15,851 | 10,402 | (65.6) | | 1.40 | | (1.37, 1.43) | 1.40 | (1.37, 1.43) |
|  | Moderate/severe impairment | 1,511 | 1,059 | (70.1) | | 1.36 | | (1.32, 1.40) | 1.36 | (1.32, 1.40) |
|  | Severe impairment | 4,907 | 3,409 | (69.5) | | 1.35 | | (1.32, 1.38) | 1.35 | (1.32, 1.38) |
|  | Very severe impairment | 711 | 439 | (61.7) | | 1.17 | | (1.10, 1.23) | 1.17 | (1.10, 1.23) |
| *District health board (DHB)* | |  |  |  | |  | |  |  |  |
|  | Northland | 5,487 | 2,584 | (47.1) | | 1.37 | | (1.32, 1.41) | 1.36 | (1.32, 1.41) |
|  | Waitemata | 9,822 | 6,484 | (66.0) | | 1.62 | | (1.58, 1.66) | 1.62 | (1.57, 1.66) |
|  | Auckland | 9,862 | 3,498 | (35.5) | | 1 | | (reference) | 1 | (reference) |
|  | Counties Manukau | 12,875 | 4,300 | (33.4) | | 1.05 | | (1.01, 1.08) | 1.05 | (1.01, 1.08) |
|  | Bay of Plenty | 10,688 | 4,405 | (41.2) | | 1.25 | | (1.22, 1.29) | 1.25 | (1.22, 1.29) |
|  | Waikato | 13,392 | 5,889 | (44.0) | | 1.38 | | (1.34, 1.42) | 1.38 | (1.34, 1.42) |
|  | Lakes | 3,520 | 1,451 | (41.2) | | 1.35 | | (1.30, 1.41) | 1.35 | (1.30, 1.41) |
|  | Tairawhiti | 1,531 | 502 | (32.8) | | 1.10 | | (1.04, 1.17) | 1.10 | (1.04, 1.17) |
|  | Taranaki | 5,199 | 1,606 | (30.9) | | 1.11 | | (1.07, 1.16) | 1.11 | (1.07, 1.16) |
|  | Whanganui | 2,716 | 1,010 | (37.2) | | 1.27 | | (1.22, 1.32) | 1.27 | (1.22, 1.33) |
|  | Hawke's Bay | 7,430 | 3,176 | (42.7) | | 1.46 | | (1.41, 1.50) | 1.46 | (1.41, 1.50) |
|  | MidCentral | 6,445 | 2,475 | (38.4) | | 1.16 | | (1.12, 1.20) | 1.16 | (1.12, 1.20) |
|  | Capital and Coast | 8,931 | 2,887 | (32.3) | | 1.03 | | (1.00, 1.07) | 1.03 | (1.00, 1.07) |
|  | Hutt Valley | 4,585 | 1,934 | (42.2) | | 1.20 | | (1.16, 1.24) | 1.20 | (1.16, 1.25) |
|  | Wairarapa | 2,254 | 691 | (30.7) | | 1.11 | | (1.06, 1.17) | 1.11 | (1.06, 1.17) |
|  | Nelson Marlborough | 6,380 | 1,809 | (28.4) | | 0.97 | | (0.94, 1.01) | 0.97 | (0.94, 1.01) |
|  | West Coast | 1,243 | 286 | (23.0) | | 0.84 | | (0.77, 0.91) | 0.84 | (0.77, 0.91) |
|  | Canterbury | 15,518 | 6,680 | (43.0) | | 1.41 | | (1.37, 1.45) | 1.41 | (1.37, 1.45) |
|  | South Canterbury | 3,135 | 1,144 | (36.5) | | 1.25 | | (1.20, 1.30) | 1.25 | (1.20, 1.30) |
|  | Southern | 12,700 | 7,131 | (56.1) | | 1.84 | | (1.79, 1.89) | 1.84 | (1.79, 1.89) |

Note: ^a^adjusted for all variables contained within Table S1.
